# Synthesis, crystal structure and Hirshfeld surface analysis of 2-{4-[(2-chloro­phen­yl)meth­yl]-3-methyl-6-oxopyridazin-1-yl}-*N*-phenyl­acetamide

**DOI:** 10.1107/S2056989024010296

**Published:** 2024-10-31

**Authors:** Hamza Assila, Younes Zaoui, Camille Kalonji Mubengayi, Walid Guerrab, Abdulsalam Alsubari, Joel T. Mague, Youssef Ramli, Mhammed Ansar

**Affiliations:** a Laboratory of Medicinal Chemistry Drug Sciences Research Center Faculty of Medicine and Pharmacy Mohammed V University in Rabat Rabat Morocco; b Laboratoire de Chimie et Biochimie, Institut Superieur des Techniques Medicales Kinshasa, Republique Democratique, du, Congo; c Laboratory of Medicinal Chemistry, Faculty of Clinical Pharmacy, 21 September University, Yemen; d Department of Chemistry, Tulane University, New Orleans, LA 70118, USA; Katholieke Universiteit Leuven, Belgium

**Keywords:** crystal structure, hydrogen bond, C—H⋯π(ring) inter­action, acetamide, pyridazine

## Abstract

In the title mol­ecule, C_20_H_18_ClN_3_O_2_, the 2-chloro­phenyl group is disordered to a small extent [occupancies 0.875 (2)/0.125 (2)]. The phenyl­acetamide moiety is nearly planar due to a weak, intra­molecular C—H⋯O hydrogen bond. In the crystal, N—H⋯O hydrogen bonds and π-stacking inter­actions between pyridazine and phenyl rings form helical chains of mol­ecules in the *b*-axis direction, which are linked by C—H⋯O hydrogen bonds and C—H⋯π(ring) inter­actions. A Hirshfeld surface analysis was performed, which showed that H⋯H, C⋯H/H⋯C and O⋯H/H⋯O inter­actions to dominate the inter­molecular contacts in the crystal.

## Abstract 2

The phenyl­acetamide moiety is nearly planar due to a weak, intra­molecular C—H⋯O hydrogen bond and its nitro­gen lone pair appears involved in N→C π bonding. In the crystal, N—H⋯O hydrogen bonds and π-stacking inter­actions between pyridazine and phenyl rings form helical chains of mol­ecules, which are linked by C—H⋯O hydrogen bonds and C—H⋯π(ring) inter­actions.

## 1. Chemical context

Various classes of heterocyclic compounds have been widely proven to exhibit diverse biological activities (Ameziane El Hassani *et al.*, 2023 ▸; Missioui *et al.*, 2022*a* ▸). Among them, pyridazin-3(2*H*)-one derivatives have emerged as one of the most studied scaffolds in recent decades (Akhtar *et al.*, 2016 ▸; Dubey & Bhosle, 2015 ▸). Known as a ‘wonder nucleus’, pyridazin-3(2*H*)-one has provided numerous derivatives with diverse pharmacological profiles. This heterocyclic compound has been shown to possess various biological activities, including anti-microbial (Özdem\?r *et al.*, 2019 ▸), anti-cancer (Bouchmaa *et al.*, 2019 ▸), butyrylcholinesterase inhibitors (Dundar *et al.*, 2019 ▸), anti-convulsant (Siddiqui *et al.*, 2020 ▸), anti-inflammatory (Boukharsa *et al.*, 2018 ▸; Zaoui *et al.*, 2021 ▸), anti-diabetic (Assila *et al.*, 2024 ▸; Boukharsa *et al.*, 2024 ▸). Acetamide derivatives, due to their wide range of activities (Missioui *et al.*, 2022*a* ▸,*b* ▸; Mortada *et al.*, 2023 ▸; Dahmani *et al.*, 2024 ▸), continue to hold significant importance as inter­mediates in organic chemistry. As a continuation of our work in synthesizing new *N*-aryl­acetamide derivatives (Guerrab *et al.*, 2021 ▸; Missioui *et al.*, 2020 ▸, 2021 ▸), and developing new pyridazine-3(2*H*)-one compounds (Zaoui *et al.*, 2022 ▸), the title compound, C_20_H_18_ClN_3_O_2_, was synthesized and its crystal structure is reported here. A Hirshfeld surface analysis was performed to analyze the inter­molecular inter­actions.
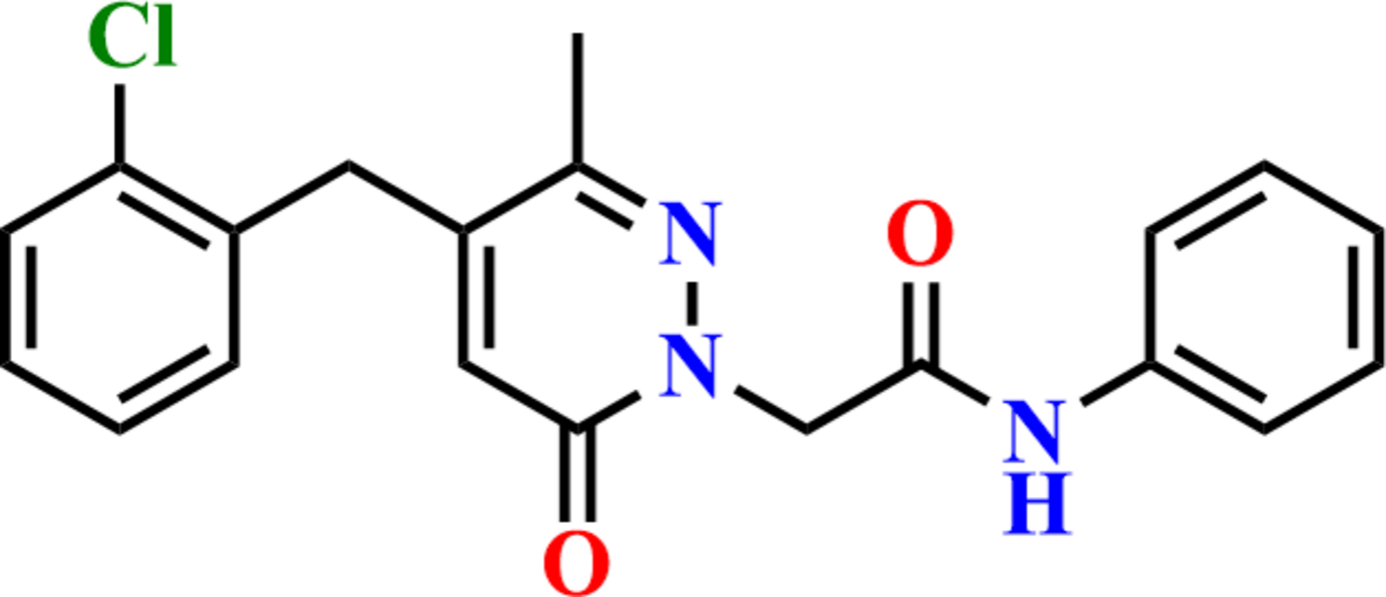


## 2. Structural commentary

**Figure 1:**
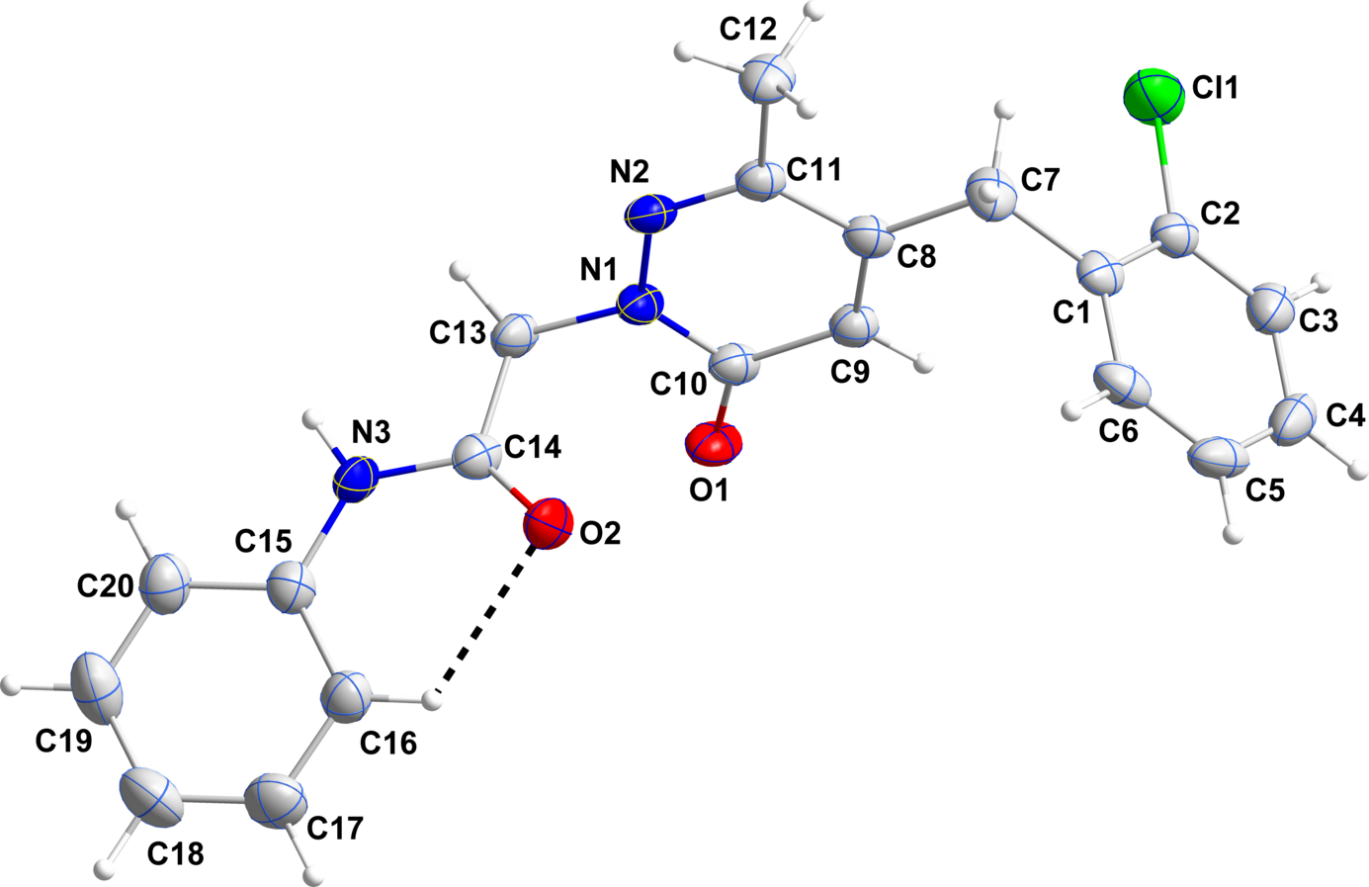
The title mol­ecule with labeling scheme and 50% probability ellipsoids. The intra­molecular C—H⋯O hydrogen bond is depicted by a dashed line. Only the major portion of the disordered 2-chloro­phenyl group is shown.

The dihedral angle between the mean planes of the C15–C20 and the pyridazine rings is 56.13 (13)° while that between the mean planes of the pyridazine and the major component of the disordered 2-chloro­phenyl rings is 80.98 (11)°. The two components of the latter ring make a dihedral angle of 4.2 (12)°. The phenyl­acetamide moiety is nearly planar [largest deviation of an atom from the mean plane is 0.003 (3) Å] due to the weak, intra­molecular C16—H16⋯O2 hydrogen bond (Table 1 ▸, Fig. 1 ▸) and the sum of the angles about N3 is 360° within experimental error. This suggests involvement of its lone pair in N→C π bonding in support of which, the N3—C14 and N3—C15 bond distances are, respectively, 1.363 (4) and 1.409 (5) Å.

## 3. Supra­molecular features

**Figure 2:**
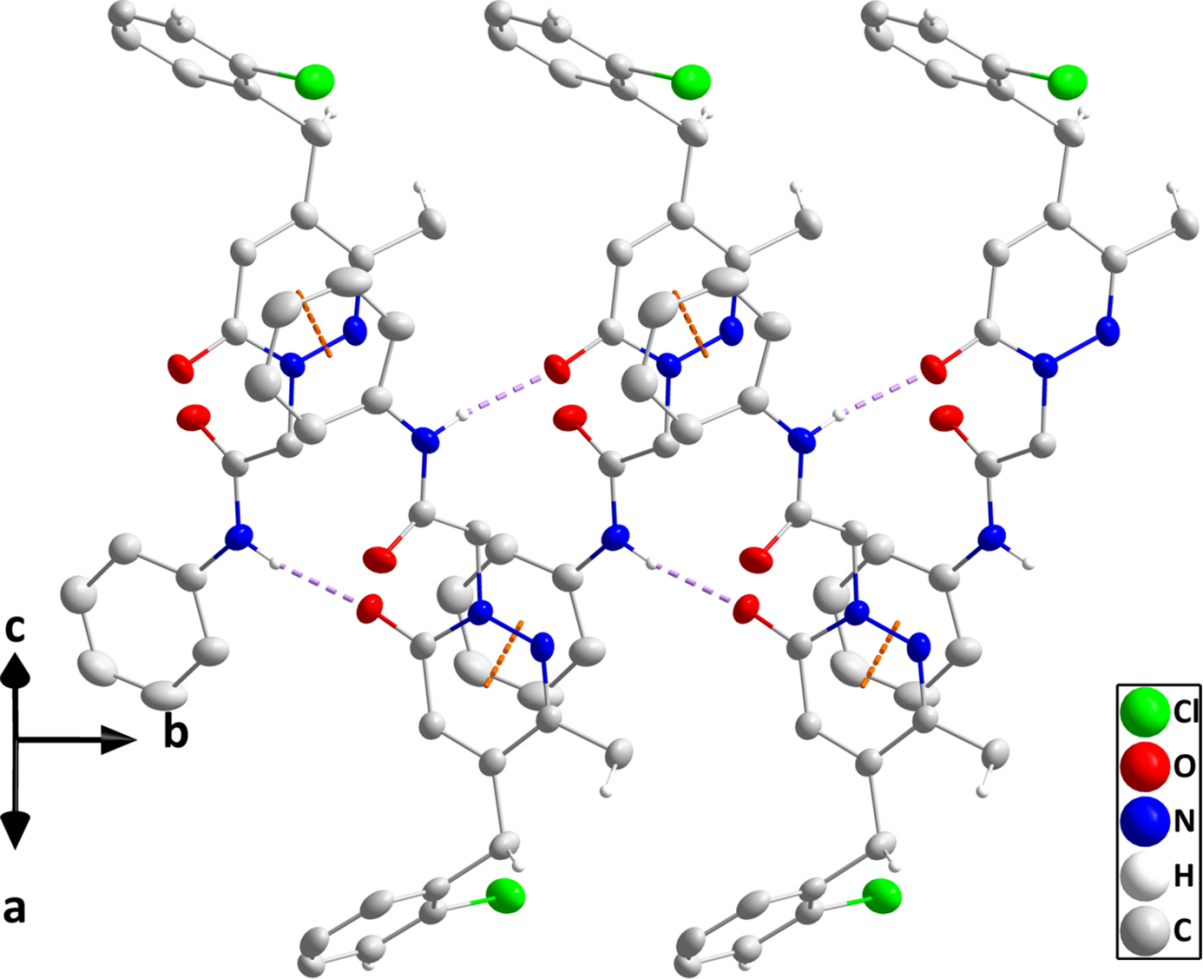
Perspective view of a portion of one chain of mol­ecules. N—H⋯O hydrogen bonds and π-stacking inter­actions are depicted, respectively, by violet and orange dashed lines. Non-inter­acting hydrogen atoms and the minor portion of the disordered 2-chloro­phenyl group are omitted for clarity.

**Figure 3:**
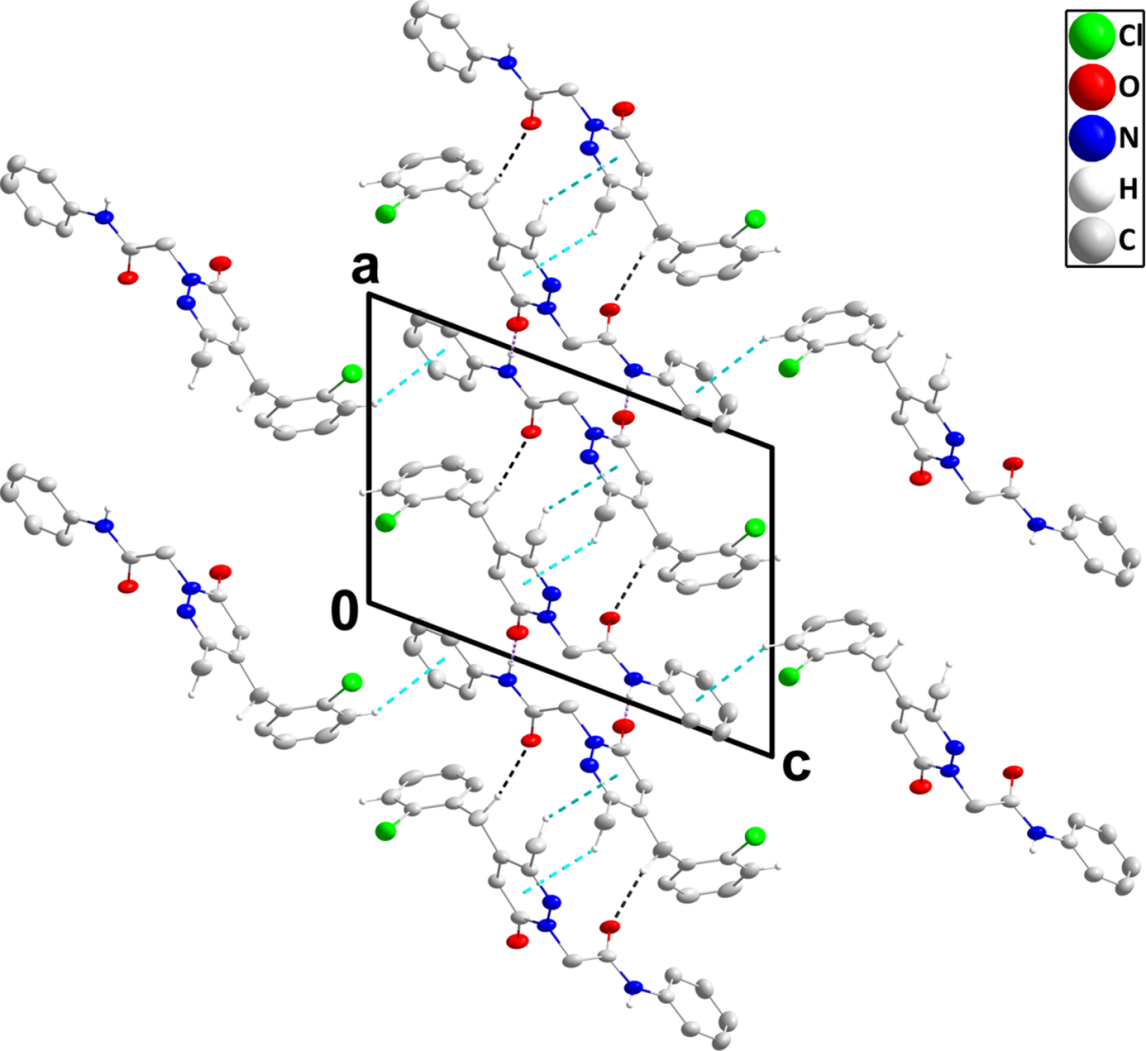
Packing viewed along the *b*-axis direction with N—H⋯O and C—H⋯O hydrogen bonds depicted, respectively, by violet and black dashed lines while C—H⋯π(ring) inter­actions are depicted by light-blue dashed lines. The π-stacking inter­actions, non-inter­acting hydrogen atoms and the minor portion of the disordered 2-chloro­phenyl group are omitted for clarity.

In the crystal, N3—H3⋯O1 hydrogen bonds and π-stacking inter­actions between pyridazine and C15–C20 rings related by the symmetry operation −*x*, *y* + 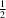, −*z* + 1 [centroid–centroid distance = 3.691 (2) Å, dihedral angle = 2.13 (18)°, slippage = 1.25 Å] form helical chains of mol­ecules extending along the *b*-axis direction (Table 1 ▸ and Fig. 2 ▸). These are connected by C7—H7*A*⋯O2 hydrogen bonds and C3—H3*A*⋯*Cg*3 and C12—H12*B*⋯*Cg*1 inter­actions (Table 1 ▸; *Cg*1 and *Cg*3 are the centroids of the C8/C9/C10/N1/N2/C11 and C15–C20 rings, respectively), forming the full three-dimensional structure (Fig. 3 ▸).

## 4. Database survey

**Figure 4:**
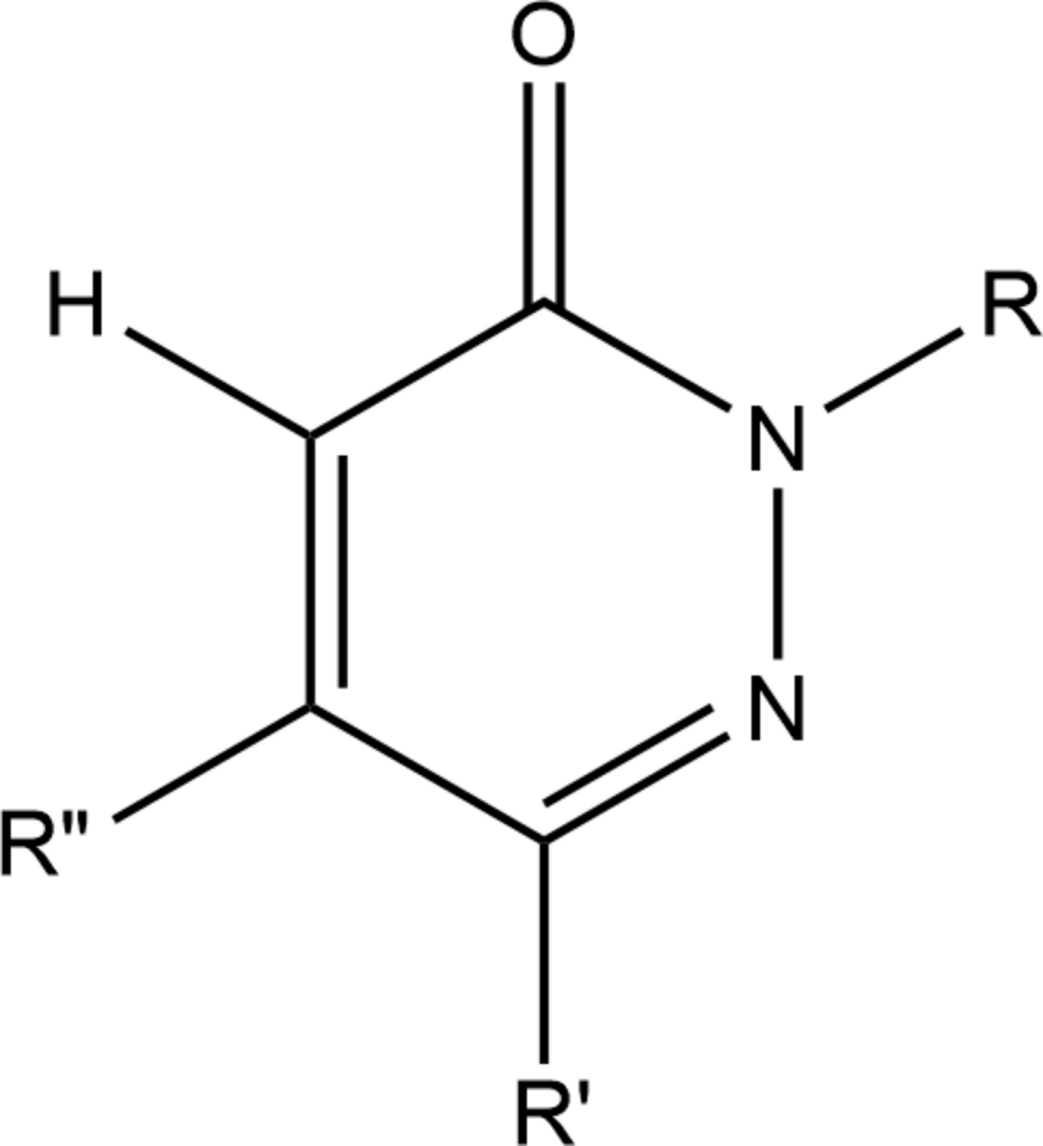
The search fragment used in the Cambridge Structural Database search.

A search of the Cambridge Structural Database (CSD, updated to June 2024; Groom *et al.*, 2016 ▸) with the fragment shown in Fig. 4 ▸ (*R* = *R*′ = *R*′′ = C) gave 15 hits of which 12 were considered similar to the title mol­ecule. The closest analog has *R* = 4-FC_6_H_4_NHC(=O)CH_2_, *R*′ = Me, *R*′′ = 2-ClC_6_H_4_ (FITXUF; Assila *et al.*, 2023 ▸) and is largely the same in all respects, even down to the disorder in the 2-chloro­phenyl group. The packing is somewhat different due to the presence of inter­molecular C—H⋯F hydrogen bonds. Among the others is a group of structures having *R*′ = *R*′′ = Ph and *R* = –CH_2_COOH (CIPTOL; Aydin *et al.*, 2007 ▸), (4-meth­yl)piperazin-1-yl-C(=O)CH_2_CH_2_– (LOBTAY; Aydin *et al.*, 2008 ▸) and (4-chloro­phen­yl)piperazin-1-yl-C(=O)CH_2_CH_2_– (QEDXXA; Aydin *et al.*, 2012 ▸). The remainder are those with *R* = –CH_2_COOEt, *R*′ = Me, *R′′* = 4-MeC_6_H_4_CH_2_– (EMOGUL; Zaoui *et al.*, 2021 ▸); *R* = –CH_2_CH_2_OH, *R*′ = Me, *R′′* = 2-ClC_6_H_4_CH_2_– (IJEMOZ; Abourichaa *et al.*, 2003 ▸); *R* = (5-(tri­fluoro­meth­yl)benzo[*d*]thia­zol-2-yl)CH_2_– (JOXVUN; Mylari *et al.*, 1992 ▸); *R* = *R′′* = Ph, *R*′ = 4-ClC_6_H_4_– (QOLLOU; Mantovani *et al.*, 2014 ▸); *R* = –CH_2_COOEt, *R*′ = Me, *R*′′ = 4-ClC_6_H_4_CH_2_– (SIQXAV; Zaoui *et al.*, 2023 ▸); *R* = –CH_2_COOEt, *R*′ = Me, *R′′* = C_6_H_5_CH_2_– (WOCGON; Zaoui *et al.*, 2019 ▸); *R* = –CH_2_COOEt, *R*′ = Me, *R′′* = 5-chloro­benzo­furan-2-yl-CH_2_– (XULSEE; Boukharsa *et al.*, 2015 ▸); *R* = –CH_2_COOEt, *R*′ = Me, *R′′* = 4-MeOC_6_H_4_CH_2_– (YAZLEU; Zaoui *et al.*, 2022 ▸). In EMOGUL, IJEMOZ and WOCGON, the pyridazine ring is planar with deviations from the mean plane by no more than 0.007 Å while in CIPTOL, XULSEE and YAZLEU the ring is more ‘ruffled’; with deviations ranging from 0.022 to 0.031 Å. The most non-planar pyridazine ring was found in QEDXAA where the largest deviation is 0.062 (2) Å. In those structures where a ring or ring system is attached to the pyridazine ring *via* a methyl­ene group, that ring is nearly perpendicular to the mean plane of the pyridazine ring as is the 2-chloro­phenyl group in the title mol­ecule. Other flexible substituents are generally rotated well out of the mean plane of the pyridazine ring. In FITXUF and CIPTOL, the primary inter­molecular inter­actions are classical hydrogen bonds (N—H⋯O and O—H⋯O, respectively), which generate chains of mol­ecules as the basic building blocks of the 3-D structures. In the others, chains of mol­ecules or chains of inversion dimers are formed in most cases by C—H⋯O hydrogen bonds with additional C—H⋯O and, in some instances, C—H⋯N hydrogen bonds serving to generate the complete 3-D structures.

## 5. Hirshfeld surface analysis

**Figure 5:**
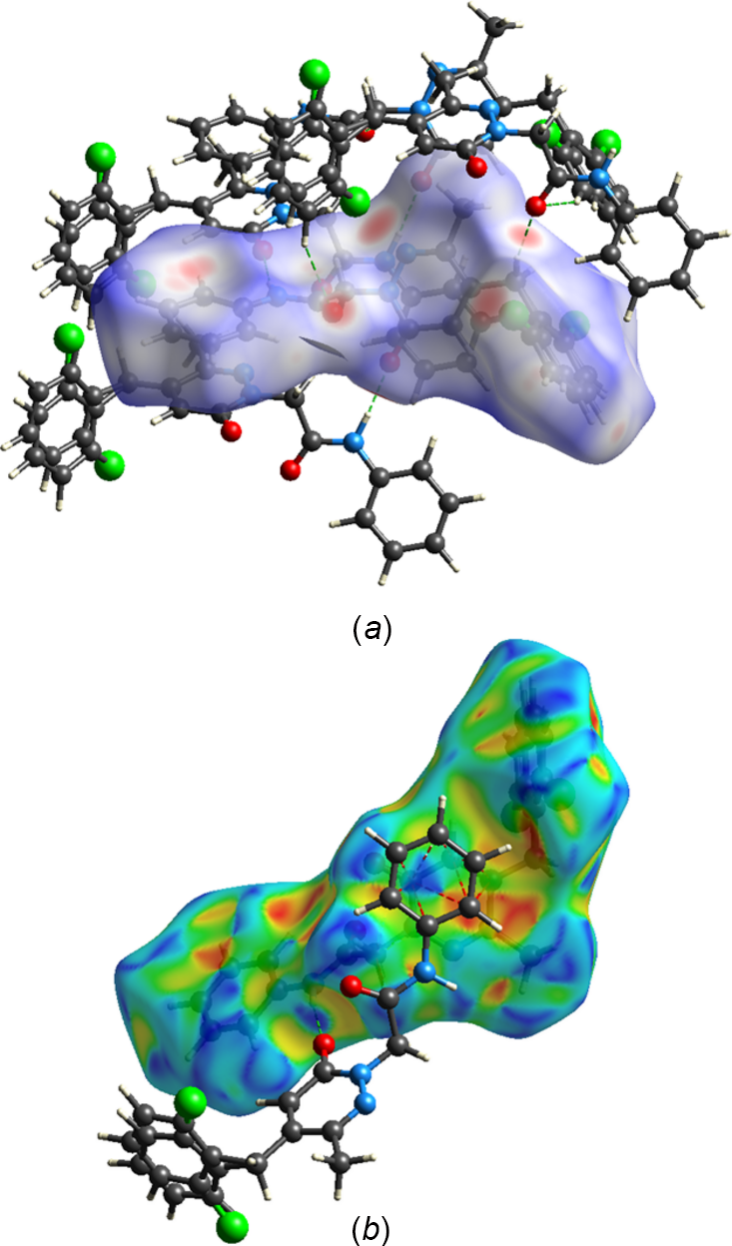
(*a*) The *d*_norm_ surface with neighboring mol­ecules showing the hydrogen bonds as green dashed lines and (*b*) the surface calculated over the shape-index function with one neighboring mol­ecule showing the π-stacking inter­actions as red dashed lines.

**Figure 6:**
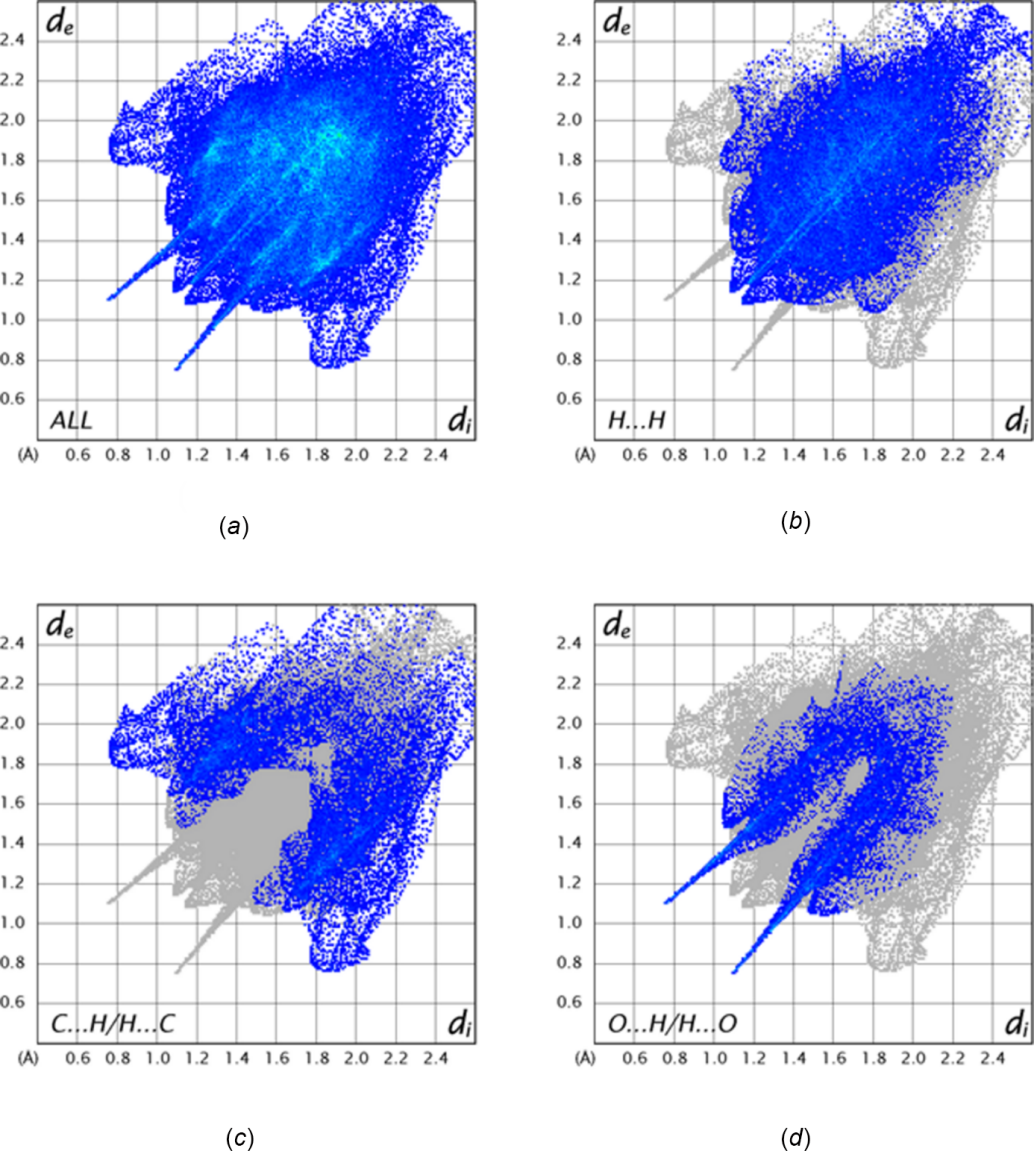
Fingerprint plots showing (*a*) all inter­molecular inter­actions and those delineated into (*b*) H⋯H inter­actions, (*c*) C⋯H/H⋯C inter­actions and (*d*) O⋯H/H⋯O inter­actions.

A Hirshfeld surface analysis of the inter­molecular inter­actions in the crystal of the title mol­ecule was performed with *Crystal­Explorer* (Spackman *et al.*, 2021 ▸) with general details of the plots produced and their inter­pretation provided in a recent publication (Tan *et al.*, 2019 ▸). Fig. 5 ▸*a* shows the *d*_norm_ surface calculated over the range −0.5868 to 1.6936 in arbitrary units with neighboring mol­ecules that are hydrogen bonded to it (green dashed lines). Fig. 5 ▸*b* shows the surface calculated over the shape function with one neighboring mol­ecule showing the π-stacking inter­action (red dashed lines). Fingerprint plots showing the major contributions to the inter­molecular inter­actions in the crystal are presented in Fig. 6 ▸. In Fig. 6 ▸*a* all these inter­actions are shown, while Fig. 6 ▸*b*–6*d* highlight the H⋯H, C⋯H/H⋯C and O⋯H/H⋯O inter­actions, respectively. The H⋯H contacts account for 43.8% of all inter­molecular inter­actions, and result from the significant hydrogen content of the mol­ecule and the fact that most of the hydrogen atoms comprise its periphery. The C⋯H/H⋯C contacts contribute 21.0% with those indicated by peaks having the highest density at *d*_e_ + *d*_i_ = 3.3 Å coming primarily from the C—H⋯π(ring) inter­actions (Table 1 ▸). The O⋯H/H⋯O inter­actions contribute 13.7% and are represented by a pair of sharp spikes having *d*_e_ + d_i_ = 2.2 Å, which can be attributed to the N—H⋯O hydrogen bonds as well as a pair of rather broad peaks at longer distances. The latter likely represent the C—H⋯O hydrogen bonds, which have a wider distribution of H⋯O distances. Other atom–atom contacts each contribute less than 10% to the overall inter­molecular inter­actions in the crystal.

## 6. Synthesis and crystallization

A mixture of the 3-benzyl­idene-4-oxo­penta­noic acid derivative (0.01 mol) and hydrazine monohydrate (0.02 mol) in 30 mL of ethanol was refluxed to produce the 5-(2-chloro­benz­yl)-6-methyl­pyridazin-3(2*H*)-one precursor. To this pyridazin-3(2*H*)-one derivative (0.01 mol), 2-chloro-*N*-phenyl­acetamide (0.01 mol), potassium bicarbonate (0.02 mol), and a small amount of BTBA (benzyl­tri­butyl­ammonium bromide) as a phase-transfer catalyst were added. The reaction mixture was stirred at room temperature for 24 h, and the reaction progress was monitored by TLC. Afterwards, 200 mL of distilled water were added, and the resulting precipitate was filtered, dried, and recrystallized from absolute acetone, yielding transparent crystals of the target compound.

Yield 90%; m.p: (461–463 K). **IR** (KBr, ν (cm^−1^): 1597 (C=O pyridazinone), 1655 (C=O acetamide), 3279 (NH amide). **^1^H NMR** [500 MHz, DMSO-*d*_6_, δ(ppm)]: 2.25 (*s*, 3H, CH_3_); 3.96 (*s*, 2H, phen­yl–CH_2_–pyridazinone); 4.80 (*s*, 2H, N–CH_2_–CO); 6.07 (*s*, 1H, pyridazinone); 6.95–7.57 (*m*, 9H, two phen­yl); 10.26 (*s*, 1H, NH). **^13^C NMR** [126 MHz, DMSO-*d*_6_, δ(ppm)]: 19.07, 35.33, 54.69, 119.53, 123.94, 126.57, 128.31, 129.35, 129.72, 130.16, 132.08, 134.03, 134.87, 139.29, 144.85, 159.86, 165.74. MS (ESI^+^): *m*/*z* = 368.11530 [*M* + H]^+^

## 7. Refinement

Crystal data, data collection and structure refinement details are summarized in Table 2 ▸. H atoms attached to carbon were placed in calculated positions and were included as riding contributions with isotropic displacement parameters tied to those of the attached atoms. That attached to nitro­gen was placed in a location derived from a difference map and refined with a DFIX 0.91 0.01 instruction. The 2-chloro­phenyl ring is disordered over two sites by an approximate 180° rotation about the C1—C7 bond and a small translation in the plane of the ring. The two rings were refined as rigid hexa­gons and additional restraints were applied to render the geometries of the two components similar. The refined ratio for the disorder is 0.875 (2)/0.125 (2).

## Supplementary Material

Crystal structure: contains datablock(s) I. DOI: 10.1107/S2056989024010296/vm2308sup1.cif

Structure factors: contains datablock(s) I. DOI: 10.1107/S2056989024010296/vm2308Isup2.hkl

Supporting information file. DOI: 10.1107/S2056989024010296/vm2308Isup3.cml

CCDC reference: 2392686

Additional supporting information:  crystallographic information; 3D view; checkCIF report

## Figures and Tables

**Table 1:** Hydrogen-bond geometry (Å, °) *Cg*1 and *Cg*3 are the centroids of the C8/C9/C10/N1/N2/C11 and C15–C20 rings, respectively.

*D*—H⋯*A*	*D*—H	H⋯*A*	*D*⋯*A*	*D*—H⋯*A*
N3—H3⋯O1^i^	0.90 (1)	1.96 (2)	2.843 (4)	168 (4)
C3—H3*A*⋯*Cg*3^ii^	0.95	2.94	3.791 (3)	149
C7—H7*A*⋯O2^iii^	0.99	2.33	3.303 (5)	167
C12—H12*B*⋯*Cg*1^iii^	0.98	2.99	3.670 (4)	128
C16—H16⋯O2	0.95	2.32	2.918 (4)	121

Symmetry codes: (i) 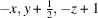; (ii) 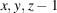; (iii) 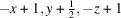.

**Table 2:** Experimental details

Crystal data	
Chemical formula	C_20_H_18_ClN_3_O_2_
*M*_r_	367.82
Crystal system, space group	Monoclinic, *P*2_1_
Temperature (K)	125
*a*, *b*, *c* (Å)	10.1898 (6), 6.7445 (4), 14.2538 (11)
β (°)	110.901 (2)
*V* (Å^3^)	915.13 (10)
*Z*	2
Radiation type	Mo *K*α
μ (mm^−1^)	0.23
Crystal size (mm)	0.35 × 0.32 × 0.05

Data collection	
Diffractometer	Bruker D8 QUEST PHOTON 3 diffractometer
Absorption correction	Numerical (*SADABS*; Krause *et al.*, 2015 ▸)
*T*_min_, *T*_max_	0.92, 0.99
No. of measured, independent and observed [*I* > 2σ(*I*)] reflections	21487, 3700, 3381
*R*_int_	0.039
(sin θ/λ)_max_ (Å^−1^)	0.626

Refinement	
*R*[*F*^2^ > 2σ(*F*^2^)], *wR*(*F*^2^), *S*	0.049, 0.111, 1.09
No. of reflections	3700
No. of parameters	237
No. of restraints	4
H-atom treatment	H atoms treated by a mixture of independent and constrained refinement
Δρ_max_, Δρ_min_ (e Å^−3^)	0.49, −0.17
Absolute structure	Flack *x* determined using 1318 quotients [(*I*^+^)−(*I*^−^)]/[(*I*^+^)+(*I*^−^)] (Parsons *et al.*, 2013 ▸)
Absolute structure parameter	0.06 (3)

Computer programs: *APEX4* and *SAINT* (Bruker, 2021 ▸), *SHELXT* (Sheldrick, 2015*a* ▸), *SHELXL2019/1* (Sheldrick, 2015*b* ▸), *DIAMOND* (Brandenburg & Putz, 2012 ▸) and *SHELXTL* (Sheldrick, 2008 ▸).

## supplementary crystallographic information

### 2-{4-[(2-Chlorophenyl)methyl]-3-methyl-6-oxopyridazin-1-yl}-N-phenylacetamide Crystal data

**Table:**

C_20_H_18_ClN_3_O_2_	*F*(000) = 384
*M**_r_* = 367.82	*D*_x_ = 1.335 Mg m^−^^3^
Monoclinic, *P*2_1_	Mo *K*α radiation, λ = 0.71073 Å
*a* = 10.1898 (6) Å	Cell parameters from 9927 reflections
*b* = 6.7445 (4) Å	θ = 3.0–26.4°
*c* = 14.2538 (11) Å	µ = 0.23 mm^−^^1^
β = 110.901 (2)°	*T* = 125 K
*V* = 915.13 (10) Å^3^	Plate, colourless
*Z* = 2	0.35 × 0.32 × 0.05 mm

### 2-{4-[(2-Chlorophenyl)methyl]-3-methyl-6-oxopyridazin-1-yl}-N-phenylacetamide Data collection

**Table:**

Bruker D8 QUEST PHOTON 3 diffractometer	3700 independent reflections
Radiation source: fine-focus sealed tube	3381 reflections with *I* > 2σ(*I*)
Graphite monochromator	*R*_int_ = 0.039
Detector resolution: 7.3910 pixels mm^-1^	θ_max_ = 26.4°, θ_min_ = 2.1°
φ and ω scans	*h* = −12→12
Absorption correction: numerical (*SADABS*; Krause *et al.*, 2015)	*k* = −8→8
*T*_min_ = 0.92, *T*_max_ = 0.99	*l* = −17→17
21487 measured reflections	

### 2-{4-[(2-Chlorophenyl)methyl]-3-methyl-6-oxopyridazin-1-yl}-N-phenylacetamide Refinement

**Table:**

Refinement on *F*^2^	Hydrogen site location: mixed
Least-squares matrix: full	H atoms treated by a mixture of independent and constrained refinement
*R*[*F*^2^ > 2σ(*F*^2^)] = 0.049	*w* = 1/[σ^2^(*F*_o_^2^) + (0.0454*P*)^2^ + 0.4761*P*] where *P* = (*F*_o_^2^ + 2*F*_c_^2^)/3
*wR*(*F*^2^) = 0.111	(Δ/σ)_max_ < 0.001
*S* = 1.09	Δρ_max_ = 0.49 e Å^−^^3^
3700 reflections	Δρ_min_ = −0.17 e Å^−^^3^
237 parameters	Absolute structure: Flack *x* determined using 1318 quotients [(*I*^+^)-(*I*^-^)]/[(*I*^+^)+(*I*^-^)] (Parsons *et al.*, 2013)
4 restraints	Absolute structure parameter: 0.06 (3)

### 2-{4-[(2-Chlorophenyl)methyl]-3-methyl-6-oxopyridazin-1-yl}-N-phenylacetamide Special details

**Table:**

Experimental. The diffraction data were obtained from 4 sets of frames, each of width 0.5° in ω or φ, collected with scan parameters determined by the "strategy" routine in *APEX4*. The scan time was 25 sec/frame.
Geometry. All esds (except the esd in the dihedral angle between two l.s. planes) are estimated using the full covariance matrix. The cell esds are taken into account individually in the estimation of esds in distances, angles and torsion angles; correlations between esds in cell parameters are only used when they are defined by crystal symmetry. An approximate (isotropic) treatment of cell esds is used for estimating esds involving l.s. planes.
Refinement. Refinement of F^2^ against ALL reflections. The weighted R-factor wR and goodness of fit S are based on F^2^, conventional R-factors R are based on F, with F set to zero for negative F^2^. The threshold expression of F^2^ > 2sigma(F^2^) is used only for calculating R-factors(gt) etc. and is not relevant to the choice of reflections for refinement. R-factors based on F^2^ are statistically about twice as large as those based on F, and R- factors based on ALL data will be even larger. H-atoms attached to carbon were placed in calculated positions (C—H = 0.95 - 0.99 Å) and were included as riding contributions with isotropic displacement parameters 1.2 - 1.5 times those of the attached atoms. That attached to nitrogen was placed in a location derived from a difference map and refined with a DFIX 0.91 0.01 instruction. The 2-chlorophenyl ring is disordered over two sites by an approximate 180° rotation about the C1—C7 bond and a small translation in the plane of the ring. The two rings were refined as rigid hexagons and additional restraints were applied to render the geometries of the two components similar. The refined ratio for the disorder is 0.875 (2)/0,125 (2).

### 2-{4-[(2-Chlorophenyl)methyl]-3-methyl-6-oxopyridazin-1-yl}-N-phenylacetamide Fractional atomic coordinates and isotropic or equivalent isotropic displacement parameters (Å^2^)

**Table:**

	*x*	*y*	*z*	*U*_iso_*/*U*_eq_	Occ. (<1)
Cl1	0.27934 (12)	0.6674 (2)	0.04167 (9)	0.0495 (3)	0.875 (2)
Cl1A	0.6147 (9)	0.3441 (14)	0.3447 (6)	0.0495 (3)	0.125 (2)
O1	0.0861 (3)	0.3089 (4)	0.3685 (2)	0.0370 (6)	
O2	0.2464 (2)	0.3392 (4)	0.5976 (2)	0.0374 (6)	
N1	0.1739 (3)	0.6043 (4)	0.4408 (2)	0.0291 (7)	
N2	0.2548 (3)	0.7696 (4)	0.4533 (2)	0.0298 (7)	
N3	0.0797 (3)	0.4603 (5)	0.6563 (2)	0.0307 (6)	
H3	0.016 (3)	0.559 (4)	0.646 (3)	0.037*	
C1	0.4556 (3)	0.4862 (3)	0.21149 (17)	0.0290 (9)	0.875 (2)
C2	0.3839 (3)	0.4761 (4)	0.10839 (17)	0.0290 (9)	0.875 (2)
C3	0.3975 (3)	0.3096 (4)	0.05509 (14)	0.0363 (10)	0.875 (2)
H3A	0.348463	0.302757	−0.015380	0.044*	0.875 (2)
C4	0.4828 (3)	0.1532 (4)	0.1049 (2)	0.0441 (12)	0.875 (2)
H4	0.492079	0.039454	0.068461	0.053*	0.875 (2)
C5	0.5545 (3)	0.1633 (3)	0.2080 (2)	0.0420 (10)	0.875 (2)
H5	0.612792	0.056389	0.242031	0.050*	0.875 (2)
C6	0.5409 (3)	0.3298 (4)	0.26129 (15)	0.0355 (10)	0.875 (2)
H6	0.589890	0.336628	0.331761	0.043*	0.875 (2)
C1A	0.418 (2)	0.519 (3)	0.1856 (11)	0.0290 (9)	0.125 (2)
C2A	0.506 (2)	0.356 (3)	0.2204 (7)	0.0290 (9)	0.125 (2)
C3A	0.508 (2)	0.205 (2)	0.1544 (14)	0.0363 (10)	0.125 (2)
H3B	0.567692	0.093525	0.178101	0.044*	0.125 (2)
C4A	0.423 (2)	0.218 (2)	0.0536 (12)	0.0441 (12)	0.125 (2)
H4A	0.424547	0.114648	0.008509	0.053*	0.125 (2)
C5A	0.336 (2)	0.381 (3)	0.0189 (6)	0.0420 (10)	0.125 (2)
H5A	0.277563	0.389180	−0.049963	0.050*	0.125 (2)
C6A	0.333 (2)	0.531 (2)	0.0849 (12)	0.0355 (10)	0.125 (2)
H6A	0.273723	0.642592	0.061157	0.043*	0.125 (2)
C7	0.4373 (4)	0.6682 (6)	0.2715 (3)	0.0377 (8)	
H7A	0.530622	0.706460	0.320000	0.045*	
H7B	0.401834	0.780294	0.224379	0.045*	
C8	0.3387 (3)	0.6365 (5)	0.3282 (2)	0.0275 (7)	
C9	0.2553 (3)	0.4749 (5)	0.3170 (3)	0.0282 (7)	
H9	0.256025	0.375222	0.270067	0.034*	
C10	0.1656 (3)	0.4513 (5)	0.3749 (3)	0.0293 (7)	
C11	0.3333 (3)	0.7870 (5)	0.3987 (2)	0.0275 (7)	
C12	0.4186 (4)	0.9741 (6)	0.4144 (3)	0.0377 (9)	
H12A	0.394636	1.045273	0.350544	0.057*	
H12B	0.518800	0.940812	0.439537	0.057*	
H12C	0.397954	1.058368	0.463472	0.057*	
C13	0.0906 (4)	0.5972 (5)	0.5049 (3)	0.0318 (8)	
H13A	−0.006953	0.558849	0.464055	0.038*	
H13B	0.087788	0.730915	0.532710	0.038*	
C14	0.1493 (3)	0.4510 (5)	0.5906 (3)	0.0293 (7)	
C15	0.0918 (3)	0.3325 (6)	0.7371 (3)	0.0308 (8)	
C16	0.1674 (4)	0.1558 (6)	0.7552 (3)	0.0356 (8)	
H16	0.221207	0.120335	0.715397	0.043*	
C17	0.1635 (4)	0.0319 (7)	0.8320 (3)	0.0452 (10)	
H17	0.214380	−0.089253	0.843960	0.054*	
C18	0.0871 (5)	0.0822 (7)	0.8909 (3)	0.0505 (11)	
H18	0.084005	−0.004487	0.942638	0.061*	
C19	0.0149 (5)	0.2595 (8)	0.8743 (3)	0.0522 (12)	
H19	−0.036243	0.295941	0.915817	0.063*	
C20	0.0162 (4)	0.3842 (7)	0.7983 (3)	0.0400 (9)	
H20	−0.034335	0.505612	0.787291	0.048*	

### 2-{4-[(2-Chlorophenyl)methyl]-3-methyl-6-oxopyridazin-1-yl}-N-phenylacetamide Atomic displacement parameters (Å^2^)

**Table:**

	*U*^11^	*U*^22^	*U*^33^	*U*^12^	*U*^13^	*U*^23^
Cl1	0.0450 (6)	0.0538 (7)	0.0516 (6)	0.0160 (6)	0.0197 (5)	0.0161 (6)
Cl1A	0.0450 (6)	0.0538 (7)	0.0516 (6)	0.0160 (6)	0.0197 (5)	0.0161 (6)
O1	0.0345 (13)	0.0240 (13)	0.0591 (16)	−0.0059 (11)	0.0247 (12)	0.0021 (12)
O2	0.0287 (13)	0.0395 (16)	0.0501 (15)	0.0115 (12)	0.0215 (11)	0.0096 (12)
N1	0.0264 (14)	0.0222 (15)	0.0441 (17)	−0.0008 (12)	0.0194 (13)	0.0030 (12)
N2	0.0280 (15)	0.0199 (14)	0.0423 (17)	0.0021 (12)	0.0137 (13)	0.0044 (13)
N3	0.0265 (14)	0.0277 (16)	0.0419 (16)	0.0062 (13)	0.0172 (13)	0.0015 (14)
C1	0.024 (2)	0.028 (2)	0.040 (2)	−0.0051 (18)	0.0176 (17)	0.0035 (19)
C2	0.0255 (19)	0.028 (2)	0.037 (2)	−0.0005 (16)	0.0149 (16)	0.0055 (19)
C3	0.039 (2)	0.032 (2)	0.046 (2)	−0.006 (2)	0.024 (2)	−0.003 (2)
C4	0.051 (3)	0.027 (2)	0.073 (3)	−0.003 (2)	0.045 (3)	−0.006 (2)
C5	0.037 (2)	0.030 (2)	0.067 (3)	0.005 (2)	0.028 (2)	0.018 (2)
C6	0.029 (2)	0.040 (3)	0.039 (2)	−0.0026 (19)	0.0138 (19)	0.015 (2)
C1A	0.024 (2)	0.028 (2)	0.040 (2)	−0.0051 (18)	0.0176 (17)	0.0035 (19)
C2A	0.0255 (19)	0.028 (2)	0.037 (2)	−0.0005 (16)	0.0149 (16)	0.0055 (19)
C3A	0.039 (2)	0.032 (2)	0.046 (2)	−0.006 (2)	0.024 (2)	−0.003 (2)
C4A	0.051 (3)	0.027 (2)	0.073 (3)	−0.003 (2)	0.045 (3)	−0.006 (2)
C5A	0.037 (2)	0.030 (2)	0.067 (3)	0.005 (2)	0.028 (2)	0.018 (2)
C6A	0.029 (2)	0.040 (3)	0.039 (2)	−0.0026 (19)	0.0138 (19)	0.015 (2)
C7	0.041 (2)	0.0331 (19)	0.046 (2)	−0.0146 (19)	0.0252 (17)	−0.0070 (19)
C8	0.0227 (15)	0.0263 (18)	0.0326 (17)	0.0000 (14)	0.0089 (13)	0.0054 (15)
C9	0.0264 (16)	0.0240 (18)	0.0350 (17)	−0.0009 (15)	0.0121 (13)	0.0015 (15)
C10	0.0233 (16)	0.0239 (17)	0.0426 (19)	0.0010 (15)	0.0139 (14)	0.0057 (16)
C11	0.0238 (16)	0.0235 (17)	0.0355 (19)	−0.0001 (14)	0.0109 (14)	0.0037 (15)
C12	0.0381 (19)	0.0272 (19)	0.053 (2)	−0.0102 (17)	0.0222 (17)	−0.0051 (18)
C13	0.0287 (17)	0.0249 (18)	0.049 (2)	0.0045 (14)	0.0221 (16)	0.0034 (15)
C14	0.0218 (15)	0.0254 (17)	0.0426 (19)	−0.0024 (15)	0.0140 (14)	0.0003 (16)
C15	0.0247 (16)	0.034 (2)	0.0339 (18)	−0.0034 (15)	0.0105 (15)	−0.0024 (15)
C16	0.0340 (18)	0.037 (2)	0.0361 (18)	0.0036 (18)	0.0130 (15)	0.0006 (18)
C17	0.045 (2)	0.046 (3)	0.040 (2)	0.0059 (19)	0.0106 (18)	0.0084 (18)
C18	0.052 (3)	0.057 (3)	0.043 (2)	−0.003 (2)	0.018 (2)	0.016 (2)
C19	0.047 (2)	0.075 (3)	0.042 (2)	0.002 (2)	0.025 (2)	0.003 (2)
C20	0.0321 (19)	0.047 (2)	0.044 (2)	0.0025 (18)	0.0175 (17)	−0.0005 (18)

### 2-{4-[(2-Chlorophenyl)methyl]-3-methyl-6-oxopyridazin-1-yl}-N-phenylacetamide Geometric parameters (Å, º)

**Table:**

Cl1—C2	1.726 (2)	C4A—H4A	0.9500
Cl1A—C2A	1.725 (3)	C5A—C6A	1.3900
O1—C10	1.238 (4)	C5A—H5A	0.9500
O2—C14	1.220 (4)	C6A—H6A	0.9500
N1—N2	1.360 (4)	C7—C8	1.512 (4)
N1—C10	1.377 (5)	C7—H7A	0.9900
N1—C13	1.454 (4)	C7—H7B	0.9900
N2—C11	1.305 (4)	C8—C9	1.356 (5)
N3—C14	1.363 (4)	C8—C11	1.444 (5)
N3—C15	1.409 (5)	C9—C10	1.443 (5)
N3—H3	0.902 (14)	C9—H9	0.9500
C1—C2	1.3900	C11—C12	1.503 (5)
C1—C6	1.3900	C12—H12A	0.9800
C1—C7	1.545 (4)	C12—H12B	0.9800
C2—C3	1.3900	C12—H12C	0.9800
C3—C4	1.3900	C13—C14	1.517 (5)
C3—H3A	0.9500	C13—H13A	0.9900
C4—C5	1.3900	C13—H13B	0.9900
C4—H4	0.9500	C15—C16	1.393 (5)
C5—C6	1.3900	C15—C20	1.397 (5)
C5—H5	0.9500	C16—C17	1.388 (6)
C6—H6	0.9500	C16—H16	0.9500
C1A—C2A	1.3900	C17—C18	1.377 (6)
C1A—C6A	1.3900	C17—H17	0.9500
C1A—C7	1.544 (5)	C18—C19	1.379 (7)
C2A—C3A	1.3900	C18—H18	0.9500
C3A—C4A	1.3900	C19—C20	1.376 (6)
C3A—H3B	0.9500	C19—H19	0.9500
C4A—C5A	1.3900	C20—H20	0.9500
			
N2—N1—C10	126.2 (3)	C1—C7—H7B	108.6
N2—N1—C13	114.0 (3)	H7A—C7—H7B	107.6
C10—N1—C13	119.7 (3)	C9—C8—C11	118.0 (3)
C11—N2—N1	117.8 (3)	C9—C8—C7	124.0 (3)
C14—N3—C15	128.2 (3)	C11—C8—C7	118.1 (3)
C14—N3—H3	116 (3)	C8—C9—C10	121.3 (3)
C15—N3—H3	115 (3)	C8—C9—H9	119.3
C2—C1—C6	120.0	C10—C9—H9	119.3
C2—C1—C7	120.2 (2)	O1—C10—N1	120.7 (3)
C6—C1—C7	119.8 (2)	O1—C10—C9	125.1 (3)
C1—C2—C3	120.0	N1—C10—C9	114.2 (3)
C1—C2—Cl1	122.29 (16)	N2—C11—C8	122.4 (3)
C3—C2—Cl1	117.70 (16)	N2—C11—C12	115.5 (3)
C4—C3—C2	120.0	C8—C11—C12	122.1 (3)
C4—C3—H3A	120.0	C11—C12—H12A	109.5
C2—C3—H3A	120.0	C11—C12—H12B	109.5
C3—C4—C5	120.0	H12A—C12—H12B	109.5
C3—C4—H4	120.0	C11—C12—H12C	109.5
C5—C4—H4	120.0	H12A—C12—H12C	109.5
C6—C5—C4	120.0	H12B—C12—H12C	109.5
C6—C5—H5	120.0	N1—C13—C14	112.1 (3)
C4—C5—H5	120.0	N1—C13—H13A	109.2
C5—C6—C1	120.0	C14—C13—H13A	109.2
C5—C6—H6	120.0	N1—C13—H13B	109.2
C1—C6—H6	120.0	C14—C13—H13B	109.2
C2A—C1A—C6A	120.0	H13A—C13—H13B	107.9
C2A—C1A—C7	110.6 (14)	O2—C14—N3	125.3 (3)
C6A—C1A—C7	129.4 (14)	O2—C14—C13	122.9 (3)
C1A—C2A—C3A	120.0	N3—C14—C13	111.8 (3)
C1A—C2A—Cl1A	119.8 (13)	C16—C15—C20	119.3 (3)
C3A—C2A—Cl1A	120.2 (13)	C16—C15—N3	123.8 (3)
C4A—C3A—C2A	120.0	C20—C15—N3	116.8 (3)
C4A—C3A—H3B	120.0	C17—C16—C15	119.5 (3)
C2A—C3A—H3B	120.0	C17—C16—H16	120.3
C3A—C4A—C5A	120.0	C15—C16—H16	120.3
C3A—C4A—H4A	120.0	C18—C17—C16	120.9 (4)
C5A—C4A—H4A	120.0	C18—C17—H17	119.5
C6A—C5A—C4A	120.0	C16—C17—H17	119.5
C6A—C5A—H5A	120.0	C17—C18—C19	119.4 (4)
C4A—C5A—H5A	120.0	C17—C18—H18	120.3
C5A—C6A—C1A	120.0	C19—C18—H18	120.3
C5A—C6A—H6A	120.0	C20—C19—C18	120.7 (4)
C1A—C6A—H6A	120.0	C20—C19—H19	119.6
C8—C7—C1A	114.4 (10)	C18—C19—H19	119.6
C8—C7—C1	114.7 (3)	C19—C20—C15	120.1 (4)
C8—C7—H7A	108.6	C19—C20—H20	120.0
C1—C7—H7A	108.6	C15—C20—H20	120.0
C8—C7—H7B	108.6		
			
C10—N1—N2—C11	−0.4 (5)	C1—C7—C8—C11	170.1 (3)
C13—N1—N2—C11	179.7 (3)	C11—C8—C9—C10	−1.1 (5)
C6—C1—C2—C3	0.0	C7—C8—C9—C10	178.7 (3)
C7—C1—C2—C3	−178.2 (3)	N2—N1—C10—O1	−178.7 (3)
C6—C1—C2—Cl1	−178.6 (2)	C13—N1—C10—O1	1.1 (5)
C7—C1—C2—Cl1	3.2 (3)	N2—N1—C10—C9	1.2 (5)
C1—C2—C3—C4	0.0	C13—N1—C10—C9	−179.0 (3)
Cl1—C2—C3—C4	178.6 (2)	C8—C9—C10—O1	179.6 (3)
C2—C3—C4—C5	0.0	C8—C9—C10—N1	−0.3 (5)
C3—C4—C5—C6	0.0	N1—N2—C11—C8	−1.3 (5)
C4—C5—C6—C1	0.0	N1—N2—C11—C12	179.0 (3)
C2—C1—C6—C5	0.0	C9—C8—C11—N2	2.0 (5)
C7—C1—C6—C5	178.2 (3)	C7—C8—C11—N2	−177.8 (3)
C6A—C1A—C2A—C3A	0.0	C9—C8—C11—C12	−178.3 (3)
C7—C1A—C2A—C3A	−178.3 (19)	C7—C8—C11—C12	1.9 (5)
C6A—C1A—C2A—Cl1A	178.2 (17)	N2—N1—C13—C14	−105.6 (3)
C7—C1A—C2A—Cl1A	−0.1 (16)	C10—N1—C13—C14	74.6 (4)
C1A—C2A—C3A—C4A	0.0	C15—N3—C14—O2	−8.2 (6)
Cl1A—C2A—C3A—C4A	−178.2 (17)	C15—N3—C14—C13	171.3 (3)
C2A—C3A—C4A—C5A	0.0	N1—C13—C14—O2	−7.1 (5)
C3A—C4A—C5A—C6A	0.0	N1—C13—C14—N3	173.5 (3)
C4A—C5A—C6A—C1A	0.0	C14—N3—C15—C16	−8.5 (6)
C2A—C1A—C6A—C5A	0.0	C14—N3—C15—C20	175.1 (3)
C7—C1A—C6A—C5A	178 (2)	C20—C15—C16—C17	1.6 (5)
C2A—C1A—C7—C8	−91.0 (12)	N3—C15—C16—C17	−174.7 (4)
C6A—C1A—C7—C8	90.9 (16)	C15—C16—C17—C18	−0.6 (6)
C2—C1—C7—C8	101.0 (3)	C16—C17—C18—C19	−0.9 (7)
C6—C1—C7—C8	−77.2 (3)	C17—C18—C19—C20	1.4 (7)
C1A—C7—C8—C9	9.9 (9)	C18—C19—C20—C15	−0.4 (6)
C1—C7—C8—C9	−9.7 (5)	C16—C15—C20—C19	−1.1 (6)
C1A—C7—C8—C11	−170.3 (8)	N3—C15—C20—C19	175.4 (4)

### 2-{4-[(2-Chlorophenyl)methyl]-3-methyl-6-oxopyridazin-1-yl}-N-phenylacetamide Hydrogen-bond geometry (Å, º)

*Cg*1 and *Cg*3 are the centroids of the C8/C9/C10/N1/N2/C11 and C15–C20 rings, respectively.

**Table:**

*D*—H···*A*	*D*—H	H···*A*	*D*···*A*	*D*—H···*A*
N3—H3···O1^i^	0.90 (1)	1.96 (2)	2.843 (4)	168 (4)
C3—H3*A*···*Cg*3^ii^	0.95	2.94	3.791 (3)	149
C7—H7*A*···O2^iii^	0.99	2.33	3.303 (5)	167
C12—H12*B*···*Cg*1^iii^	0.98	2.99	3.670 (4)	128
C16—H16···O2	0.95	2.32	2.918 (4)	121

Symmetry codes: (i) −*x*, *y*+1/2, −*z*+1; (ii) *x*, *y*, *z*−1; (iii) −*x*+1, *y*+1/2, −*z*+1.
